# Analyzing Genome-Wide Association Study Dataset Highlights Immune Pathways in Lip Bone Mineral Density

**DOI:** 10.3389/fgene.2020.00004

**Published:** 2020-03-10

**Authors:** Xiaodong Liu, Yiwei Zhang, Jun Tian, Feng Gao

**Affiliations:** Department of Trauma and Emergency Surgeon, The Second Affiliated Hospital, Harbin Medical University, Harbin, China

**Keywords:** osteoporosis, bone mineral density, genome-wide association studies, pathway analysis, immune pathways

## Abstract

Osteoporosis is a common complex human disease. Until now, large-scale genome-wide association studies (GWAS) using single genetic variant have reported some novel osteoporosis susceptibility variants. However, these risk variants only explain a small proportion of osteoporosis genetic risk, and most genetic risk is largely unknown. Interestingly, the pathway analysis method has been used in investigation of osteoporosis mechanisms and reported some novel pathways. Until now, it remains unclear whether there are other risk pathways involved in BMD. Here, we selected a lip BMD GWAS with 301,019 SNPs in 5,858 Europeans, and conducted a gene-based analysis (SET SCREEN TEST) and a pathway-based analysis (WebGestalt). On the gene level, BMD susceptibility genes reported by previous GWAS were identified to be the top 10 significant signals. On the pathway level, we identified 27 significant KEGG pathways. Three immune pathways including T cell receptor signaling pathway (hsa04660), complement and coagulation cascades (hsa04610), and intestinal immune network for IgA production (hsa04672) are ranked the top three significant signals. Evidence from the PubMed and Google Scholar databases further supports our findings. In summary, our findings provide complementary information to these nine risk pathways.

## Introduction

Osteoporosis is a complex disease with reduced bone mineral density (BMD) and increased fracture risk (Wei et al., 2016). Until now, large-scale genome-wide association studies (GWAS) based on single genetic variant have reported some novel osteoporosis susceptibility variants (Guo et al., 2010; Richards et al., 2012; Wei et al., 2016). These osteoporosis genetic variants include rs2062375, rs13182402, rs7605378, rs12775980, rs494453, and rs784288 (Guo et al., 2010; Hsu et al., 2010; Kou et al., 2011; Hwang et al., 2013; Taylor et al., 2016). Meanwhile, some BMD related genetic variants were also identified (Kemp et al., 2017; Kim, 2018; Styrkarsdottir et al., 2019). In 2017, Kemp et al. performed a GWAS of heel BMD using 142,487 individuals from the UK Biobank (Kemp et al., 2017). They identified 307 conditionally independent genetic variants with genome-wide significance at 203 loci including 153 novel loci (Kemp et al., 2017). In 2018, Kim et al. conducted a GWAS of heel BMD using the data from UK Biobank, and identified 1,362 independent genetic variants with genome-wide significance around 899 loci including 613 novel loci (Kim, 2018). In 2019, Styrkarsdottir et al. performed a GWAS of hip and lumbar spine BMD, and identified 13 independent genetic variants at 12 loci (Styrkarsdottir et al., 2019).

However, these risk variants only explain a small proportion of osteoporosis genetic risk, and most genetic risk is largely unknown (Wei et al., 2016). Considering the limitations of GWAS on single genetic variant level, some improved method named pathway analysis of GWAS dataset or gene set analysis has been proposed and widely used in multiple human complex diseases (Eleftherohorinou et al., 2009; Hong et al., 2010; Liu et al., 2012; Holmans et al., 2013; Liu et al., 2013; Liu et al., 2014; Quan et al., 2015; Jiang et al., 2017; Liu et al., 2017).

Interestingly, the pathway analysis method has also been used in investigation of osteoporosis mechanisms and reported some novel pathways (Zhang et al., 2010; Lee et al., 2012; Wei et al., 2016). In addition to these risk pathways above, it remains unclear whether there are other risk pathways involved in BMD. Hence, we conducted a pathway analysis of hip BMD GWAS with 301,019 SNPs in 5,858 Europeans using a published gene-based analysis method (SET SCREEN TEST) (Moskvina et al., 2011) and a pathway-based analysis method (WebGestalt) (Zhang et al., 2005).

## Materials and Methods

### The BMD GWAS Dataset

This study included 5,861 Icelandic persons, and 5,858 of 5,861 persons had measurements of hip bone mineral density (Styrkarsdottir et al., 2008). For each DNA sample, the Infinium HumanHap300 or the HumanCNV370 SNP chip (Illumina) was used to genotype a total of 317,503 genetic variants (Styrkarsdottir et al., 2008). After quality control, the GWAS dataset finally included 301,019 common genetic variants (Styrkarsdottir et al., 2008). In order to evaluate the association between each genetic variant and BMD, a linear regression method was utilized (Styrkarsdottir et al., 2008). Here, we used the summary results from this BMD GWAS (Styrkarsdottir et al., 2008). More detailed results are described in the original study (Styrkarsdottir et al., 2008).

### Gene-Based Testing for BMD GWAS by a Meta-Analysis Method

We selected a set screen test method implemented in PLINK to perform a gene-based test of the whole GWAS dataset (Moskvina et al., 2011). The method could combine all *P* values from all the genetic variants in each corresponding gene by an approximate Fisher’s test and could also adjust for the linkage disequilibrium (LD) (Moskvina et al., 2011).

For all genetic variants in a specific gene, if all these genetic variants are independent, the Fisher’s statistic could be calculated by

$$x_{0}^{2}=-2\sum_{i=1}^{N}\ln(p_{i})$$

where *N* is the number of selected genetic variants in a specific gene and *p_i_* (*i* = 1, …, N) is the significance level about the association each genetic variant with BMD, *x*_0_^2^ follows a chi-square distribution with the freedom degrees = 2*N* (Moskvina et al., 2011).

If the selected genetic variants are not independent,

$$x^{2}=-2\sum_{i=1}^{N}\ln(p_{i})*4N/\sigma^{2}$$

$$\sigma^{2}=4N+\sum_{i=1}^{N-1}\sum_{j=I+1}^{N}\mathrm{cov}(-2\text{l}(p_{i}),-2ln(p_{j}))$$

$$\mathrm{cov}(-2\ln(p_{i}),-2\ln(p_{j}))=p_{ij}(3.25+0.75p_{ij})$$

where *x* ^2^ follows the central chi-square distribution with 8*N* ^2^/*σ* ^2^ as degrees of freedom (Moskvina et al., 2011). Here, the LD information is from the HapMap CEU population.

### Pathway-Based Testing for BMD GWAS Dataset

Here, we conducted a KEGG pathway analysis of the BMD risk genes using WebGestalt (Zhang et al., 2005). The enrichment analysis was performed using the hypergeometric test (Zhang et al., 2005). Here, we selected the entire entrez gene set as the reference gene assuming including *N* genes. We first assume that we have identified *S* BMD risk genes using the gene-base test. For a given pathway, we further assume that this pathway includes *m* genes and *K* BMD risk genes. The significant levels observing *K* BMD risk genes in a specific pathway is

$$P=1-\sum_{i=0}^{K}\frac{\left(\overset{S}{\underset{i}{}})(\overset{N-S}{\underset{m-i}{}}\right)}{\left(\overset{N}{\underset{m}{}}\right)}$$

Meanwhile, we limited the pathway analysis using the KEGG pathways with at least 20 and at most 300 genes. A false discovery rate (FDR) method was used to adjust the original significance levels. Here, we define a pathway with adjusted *P* < 0.01 to be statistically significant.

## Results

### Gene-Based Test for BMD GWAS Dataset

Using the gene-based test, several BMD susceptibility genes reported by previous GWAS were among the top 10 significant signals, which included CKAP5 (the most significant signal with *P* = 4.03E-07) (Styrkarsdottir et al., 2009), LRP4 (the most significant signal with *P* = 6.72E-05) (Styrkarsdottir et al., 2009), and C6orf97 (the 6^th^ significant signal with *P* = 1.04E-04) (Styrkarsdottir et al., 2009). Meanwhile, we identified other new BMD susceptibility genes, such as F2 (the second significant signal with *P* = 1.58E-05), LALBA (the 4^th^ significant signal with *P* = 6.86E-05), and CPN2 (the 5^th^ significant signal with *P* = 7.21E-05). Detailed information about the 5% significant genes is described in **Supplementary Table 1**.

### Pathway-Based Test for BMD GWAS Dataset

We used the top 5% significant signals (14,008*5% = 700 genes with *P* < 0.04239) from the gene-based test for following pathway analysis. We identified 27 significant KEGG pathways (adjusted *P* < 0.01) (**Table 1**). Based on the function classifications, these pathways are mainly related to immunity, cellular processes, environment, infection, cardiovascular diseases, metabolism, and circulation. The top three significant signals are related with immune system including T cell receptor signaling pathway (hsa04660), complement and coagulation cascades (hsa04610), and intestinal immune network for IgA production (hsa04672) as described in **Table 1**. The detailed information is provided in **Supplementary Table 2**.

**Table 1:** Significant KEGG pathways with *P* < 0.01 by pathway-based analysis of BMD GWAS.

Classification	Pathway ID	Pathway Name	C	O	E	R	rawP	adjP
Immune system	hsa04660	T cell receptor signaling pathway	108	9	1.61	5.58	3.68E-05	8.00E-04
Immune system	hsa04610	Complement and coagulation cascades	69	7	1.03	6.79	7.76E-05	8.00E-04
Immune system	hsa04672	Intestinal immune network for IgA production	48	6	0.72	8.37	7.80E-05	8.00E-04
Cardiovascular diseases	hsa05414	Dilated cardiomyopathy	90	8	1.34	5.95	6.26E-05	8.00E-04
Infectious diseases: parasitic	hsa05146	Amoebiasis	106	8	1.58	5.05	2.00E-04	1.30E-03
Metabolism	hsa00564	Glycerophospholipid metabolism	80	7	1.19	5.86	2.00E-04	1.30E-03
Environmental information processing	hsa04310	Wnt signaling pathway	150	9	2.24	4.02	4.00E-04	2.30E-03
Infectious diseases: bacterial	hsa05120	Epithelial cell signaling in *Helicobacter pylori* infection	68	6	1.02	5.91	5.00E-04	2.60E-03
Circulatory system	hsa04972	Pancreatic secretion	101	7	1.51	4.64	8.00E-04	3.50E-03
Infectious diseases: parasitic	hsa05145	Toxoplasmosis	132	8	1.97	4.06	9.00E-04	3.50E-03
Cellular processes	hsa04144	Endocytosis	201	10	3	3.33	1.00E-03	3.50E-03
Endocrine system	hsa04916	Melanogenesis	101	7	1.51	4.64	8.00E-04	3.50E-03
Infectious diseases: viral	hsa05160	Hepatitis C	134	8	2	4	1.00E-03	3.50E-03
Cellular processes	hsa04146	Peroxisome	79	6	1.18	5.09	1.20E-03	3.90E-03
Cardiovascular diseases	hsa05410	Hypertrophic cardiomyopathy (HCM)	83	6	1.24	4.84	1.50E-03	4.30E-03
Cellular processes	hsa04114	Oocyte meiosis	112	7	1.67	4.19	1.50E-03	4.30E-03
Immune system	hsa04621	NOD-like receptor signaling pathway	58	5	0.87	5.77	1.70E-03	4.40E-03
Environmental information processing	hsa04512	ECM-receptor interaction	85	6	1.27	4.73	1.70E-03	4.40E-03
Environmental information processing	hsa04630	Jak-STAT signaling pathway	155	8	2.31	3.46	2.40E-03	5.90E-03
Environmental information processing	hsa04080	Neuroactive ligand-receptor interaction	272	11	4.06	2.71	2.80E-03	6.60E-03
Genetic information processing	hsa03040	Spliceosome	127	7	1.9	3.69	3.10E-03	6.70E-03
Metabolism	hsa00230	Purine metabolism	162	8	2.42	3.31	3.10E-03	6.70E-03
Cellular processes	hsa04510	Focal adhesion	200	9	2.99	3.01	3.30E-03	6.90E-03
Environmental information processing	hsa04514	Cell adhesion molecules (CAMs)	133	7	1.99	3.52	4.00E-03	7.80E-03
Circulatory system	hsa04976	Bile secretion	71	5	1.06	4.72	4.20E-03	7.80E-03
Immune system	hsa04622	RIG-I-like receptor signaling pathway	71	5	1.06	4.72	4.20E-03	7.80E-03
Cellular processes	hsa04810	Regulation of actin cytoskeleton	213	9	3.18	2.83	5.00E-03	9.00E-03

C, number of reference genes in the category; O, number of genes in the gene set and also in the category; E, expected number in the category; R, ratio of enrichment; rawP, p value from hypergeometric test; adjP, p value adjusted by the multiple test adjustment.

### Verification by PubMed and Google Scholar Literature Search

We further verified these findings from pathway analysis using publicly available literatures in PubMed and Google Scholar databases. Interestingly, growing evidence supports the involvement of dilated cardiomyopathy and hypertrophic cardiomyopathy, T cell receptor signaling pathway, wnt signaling pathway, and regulation of actin cytoskeleton in MBD. More detailed information is described in **Table 2**.

**Table 2:** Literature evidences supporting that genes in measles pathway are associated with bone mineral density or osteoporosis.

Pathway	Supporting evidence	Ref
Dilated cardiomyopathy and hypertrophic cardiomyopathy	Evidence has also shown that both Dilated cardiomyopathy and Hypertrophic cardiomyopathy could result in heart failure (Olson et al., 1998; Li et al., 2006). Cross-sectional studies have shown that more than 50% of patients with congestive heart failure (CHF) have decreased bone mineral density (BMD) (Frost et al., 2007). Heart failure (HF) is associated with several factors that contribute to both reduced bone mineral density and increased risk of osteoporosis-related fractures (Lyons et al., 2011).	(Olson et al., 1998; Li et al., 2006; Frost et al., 2007; Lyons et al., 2011)
T cell receptor signaling pathway	T lymphocytes and their products act as key regulators of osteoclast formation, life span, and activity. This review presents this understanding of the process of T lymphocytes and their products mediating osteoporosis and explores some of the most recent findings and hypotheses to explain their action in bone (Zhao et al., 2009). Our results show that activated T cells can regulate systemic and local bone loss through OPGL. In summary, activated T cells produce OPGL and can directly trigger osteoclastogenesis *in vitro*; activated T cells from ctla4-/- mice have a destructive effect on bone mineral density *in vivo* that can be reversed through inhibition of OPGL; and inhibition of OPGL through OPG can completely prevent bone and cartilage loss in a T-cell-dependent arthritis model (Kong et al., 1999).	(Kong et al., 1999; Zhao et al., 2009)
Wnt signaling pathway	Wnt signaling pathway regulates bone mineral density (BMD) through the lipoprotein receptor-related protein (LRP) 5, a receptor of this signaling. Genetic variations at the *LRP5* gene locus are associated with osteoporosis. These data suggest that genetic variations in Wnt signaling genes may affect the pathogenesis of osteoporosis (Perez-Castrillon et al., 2009). Wnt signaling has emerged to play major roles in almost all aspects of skeletal development and homeostasis. Wnt signaling has become a focal point of intensive studies in skeletal development and disease. Promising effective therapeutic agents for bone diseases are being developed by targeting the Wnt signaling pathway (Regard et al., 2012). We identified 56 loci (32 new) associated with BMD at genome-wide significance (*P* < 5 × 10^−8^). Several of these factors cluster within the RANK-RANKL-OPG, mesenchymal stem cell differentiation, endochondral ossification, and Wnt signaling pathways (Estrada et al., 2012).	(Perez-Castrillon et al., 2009; Estrada et al., 2012; Regard et al., 2012)
Regulation of actin cytoskeleton	The focal adhesion, the actin cytoskeleton and cell-cycle are connected pathways (Zintzaras et al., 2011). Data from 211 studies that investigated the association between BMD and gene variants involved in these pathways were catalogued in a web-based information system and analyzed (Zintzaras et al., 2011). The results showed that genes in these three pathways are implicated in the pathogenesis of low BMD (Zintzaras et al., 2011). Genome-wide linkage studies have highlighted the chromosomal region 3p14-p22 as a quantitative trait locus for BMD (Mullin et al., 2013). The *FLNB* gene, which is thought to have a role in cytoskeletal actin dynamics, is located within this chromosomal region and presents as a strong candidate for BMD regulation (Mullin et al., 2013). Mullin et al. identified significant associations between SNPs in the *FLNB* gene and BMD in Caucasian women (Mullin et al., 2013).	(Zintzaras et al., 2011; Mullin et al., 2013)

## Discussion

Until now, pathway analysis of BMD GWAS datasets has identified several risk pathways. However, most genetic variants, risk genes, and genetic pathways influencing osteoporosis are unknown. In order to identify novel BMD risk pathways, we conducted a pathway analysis of hip BMD GWAS with 301,019 genetic variants in 5,858 Europeans using the meta-analysis method (SET SCREEN TEST) in PLINK (Moskvina et al., 2011) and a pathway-based analysis method (WebGestalt) (Zhang et al., 2005).

On the gene level, we confirmed previous identified BMD susceptibility genes such as *CKAP5* (Styrkarsdottir et al., 2009), *LRP4* (Styrkarsdottir et al., 2009), and *C6orf97* (Styrkarsdottir et al., 2009). All these genes are ranked top 10 significant signals. We also identified some new BMD susceptibility genes, which were significantly associated with BMD with *P* < 0.0001. Take the second significant signal *F2* for example, *F2* could encode coagulation factor II, thrombin (Sato et al., 2016). Pro-thrombin is cut to generate thrombin in the coagulation cascade, and further reduce the blood loss (Sato et al., 2016). It is common that procoagulant state could active thrombin in bone fracture sites (Sato et al., 2016). Take *CPN2* for example, two genetic variants rs11711157 and rs3732477 near *CPN2* have been reported to be associated with BMD in Koreans (Kim et al., 2013). These findings suggest that the meta-analysis method (SET SCREEN TEST) is effective to identify BMD susceptibility genes.

On the pathway level, we identified 27 significant KEGG pathways. Three immune pathways including T cell receptor signaling pathway (hsa04660), complement and coagulation cascades (hsa04610), and intestinal immune network for IgA production (hsa04672) are ranked the top three significant signals. In addition, we also identified other kinks of pathways associated with cellular processes, environmental information processing, infectious diseases, cardiovascular diseases, metabolism, and circulatory system, are also identified. In order to further evaluate the potential roles of these newly identified risk pathways, we conducted a comprehensive literature review. Interestingly, growing evidence from the PubMed and Google Scholar databases further supports the involvement of dilated cardiomyopathy, hypertrophic cardiomyopathy, T cell receptor signaling pathway, wnt signaling pathway, and regulation of actin cytoskeleton in MBD, as provided in **Table 2**.

In a recent study, Guo et al. conducted a pathway and network analysis of genes related to osteoporosis (Guo et al., 2019). They first retrieved 94 osteoporosis genes from PubMed (Guo et al., 2019). They further conducted an enrichment analysis, and found that these osteoporosis genes were significantly enriched in biological processes related to bone metabolism and the immune system (Guo et al., 2019). Take wnt signaling pathway for example, we identified wnt signaling to be the 7^th^ significant pathway, as provided in **Table 1**. Interestingly, Guo et al. identified wnt signaling to be the second significant pathway with *P* = 3.71 × 10^−13^ (Guo et al., 2019). Hence, our findings are consistent with recent findings.

Our study has a main difference with previous studies. In recent years, most studies focus on the single genetic variant associated with BMD, osteoporosis, or fracture (Kemp et al., 2017; Kim, 2018; Styrkarsdottir et al., 2019). However, these genetic variants only explain a small proportion of osteoporosis genetic risk (Wei et al., 2016). Hence, pathway analysis of GWAS dataset may overcome the limitations of single genetic variant. However, our current study still has some limitations. First, the sample size is relatively small and lacks validation cohort to test the robustness of these findings. In future study, we will further select other BMD GWAS datasets with large-scale sample size to demonstrate our findings. Second, we only selected one gene based method and one pathway based method. In future, we will verify our findings using other gene and pathway based methods. Third, we selected the top 5% significant signals from the gene-based test for following pathway analysis, as did in recent studies (Yoon et al., 2018; Ierodiakonou et al., 2019; White et al., 2019). Using this strategy, we selected 700 BMD risk genes with *P* < 0.04239. In fact, there are a total of 813 BMD risk genes with *P* < 0.05. Hence, our current pathway analysis findings are consistent with those using 813 BMD risk genes (data now shown). However, selection of different thresholds may have different findings, as described in recent studies (Yoon et al., 2018; Ierodiakonou et al., 2019; White et al., 2019).

In summary, two studies reported regulation-of-autophagy and other eight significant pathways (Zhang et al., 2010; Lee et al., 2012). Our findings provide complementary information to these nine risk pathways. Meanwhile, future studies using large-scale sample sizes should further verify our findings.

## Data Availability Statement

Publicly available datasets were analyzed in this study. This data can be found here: https://www.nejm.org/doi/10.1056/NEJMoa0801197. 

## Author Contributions

FG conceived and initiated the project. FG and XL analyzed the data. All authors wrote and reviewed the manuscript. 

## Funding

This work was supported by funding from Heilongjiang Natural Science Foundation (Grant No. H2016025).

## Conflict of Interest

The authors declare that the research was conducted in the absence of any commercial or financial relationships that could be construed as a potential conflict of interest.
