# Supplementary material for: Analyzing Genome-Wide Association Study Dataset Highlights Immune Pathways in Lip Bone Mineral Density
Source: Front Genet. 2020 Mar 10;11:4. doi: 10.3389/fgene.2020.00004 (PMC7077504; doi:10.3389/fgene.2020.00004)
Supplement: Supplementary file 2 [file Table_1.doc]

Supplementary Table 1, The detailed genes in significant KEGG pathways

| Pathway ID | Pathway Name | Genes |
| --- | --- | --- |
| hsa04660 | T cell receptor signaling pathway | TNF CD8B PLCG1 MAP3K14 RELA NFATC1 PAK4 NFKBIA CD3G |
| hsa04610 | Complement and coagulation cascades | MBL2 PLG F2 SERPINA1 C8A A2M C3AR1 |
| hsa04672 | Intestinal immune network for IgA production | CCL27 ITGB7 MAP3K14 IL15 CCL25 TNFSF13B |
| hsa05414 | Dilated cardiomyopathy | TNF ITGB7 ADCY4 ITGA8 ITGA7 ADCY6 TNNT2 LMNA |
| hsa05146 | Amoebiasis | TNF SERPINB10 COL11A1 RELA C8A IL12B SERPINB2 LAMB4 |
| hsa00564 | Glycerophospholipid metabolism | CDS2 GPD1L PLA2G6 LPCAT2 PLA2G12B AGPAT6 AGPAT1 |
| hsa04310 | Wnt signaling pathway | EP300 PPP2R5A SFRP4 CAMK2B WNT2 WNT16 CCND2 NFATC1 WNT9B |
| hsa05120 | Epithelial cell signaling in Helicobacter pylori infection | PLCG1 MAP3K14 ATP6V1H RELA ATP6V1A NFKBIA |
| hsa04972 | Pancreatic secretion | CPA2 ADCY6 RAB8A ADCY4 CLCA4 PLA2G6 PLA2G12B |
| hsa05145 | Toxoplasmosis | TNF NFKBIA RELA PLA2G6 PLA2G12B IL12B IL10RA LAMB4 |
| hsa04144 | Endocytosis | AP2A2 CHMP4A AP2A1 SMAD7 VPS4A IL2RB FAM125A GIT2 EEA1 ARFGAP2 |
| hsa04916 | Melanogenesis | EP300 ADCY6 WNT16 ADCY4 CAMK2B WNT9B WNT2 |
| hsa05160 | Hepatitis C | TNF RNASEL ARAF NR1H3 RELA CLDN14 NFKBIA CLDN19 |
| hsa04146 | Peroxisome | HAO2 AMACR PEX11G AGPS PEX6 XDH |
| hsa05410 | Hypertrophic cardiomyopathy (HCM) | TNF TNNT2 ITGB7 ITGA8 ITGA7 LMNA |
| hsa04114 | Oocyte meiosis | ADCY6 ADCY4 PPP2R5A YWHAH ESPL1 CAMK2B CPEB1 |
| hsa04621 | NOD-like receptor signaling pathway | TNF RELA NLRP3 NFKBIA NLRC4 |
| hsa04512 | ECM-receptor interaction | ITGB7 COL11A1 ITGA8 ITGA7 COL6A1 LAMB4 |
| hsa04630 | Jak-STAT signaling pathway | EP300 IL15 CLCF1 CCND2 IL2RB IL12B IL10RA SOCS5 |
| hsa04080 | Neuroactive ligand-receptor interaction | GRIA1 F2 CRHR2 P2RY6 CHRNA10 ADRA1D S1PR3 PLG FPR3 GPR50 C3AR1 |
| hsa03040 | Spliceosome | DHX8 LSM4 CDC40 EFTUD2 MAGOH PUF60 CDC5L |
| hsa00230 | Purine metabolism | POLR2C POLR3D ADCY4 POLR2L NT5C1A ADCY6 APRT XDH |
| hsa04510 | Focal adhesion | ITGB7 ITGA8 ITGA7 COL6A1 FLNA COL11A1 CCND2 PAK4 LAMB4 |
| hsa04514 | Cell adhesion molecules (CAMs) | CD8B ITGB7 MAG CLDN14 ITGA8 CD22 CLDN19 |
| hsa04976 | Bile secretion | ADCY6 ABCC2 ADCY4 HMGCR AQP8 |
| hsa04622 | RIG-I-like receptor signaling pathway | TNF RNF125 RELA IL12B NFKBIA |
| hsa04810 | Regulation of actin cytoskeleton | GNG12 F2 ITGB7 ARAF WASF1 ITGA8 ITGA7 FGF17 PAK4 |
